# 3-[Bis(dimethyl­amino)­methyl­ene]-1,1-diphenyl­urea

**DOI:** 10.1107/S160053681204127X

**Published:** 2012-10-06

**Authors:** Ioannis Tiritiris

**Affiliations:** a Fakultät Chemie/Organische Chemie, Hochschule Aalen, Beethovenstrasse 1, D-73430 Aalen, Germany

## Abstract

In the title compound, C_18_H_22_N_4_O, the C=N and C—N bond lengths in the CN_3_ unit are 1.3179 (11), 1.3551 (11) and 1.3737 (11) Å, indicating double- and single-bond character, respectively. The N—C—N angles are 115.91 (8), 118.20 (8) and 125.69 (8), showing a deviation of the CN_3_ plane from an ideal trigonal–planar geometry. The bonds between the N atoms and the terminal C-methyl groups all have values close to a typical single bond [1.4529 (12)–1.4624 (12) Å]. The dihedral angle between the phenyl rings is 79.63 (4)°. In the crystal, the mol­ecules are connected *via* weak C—H⋯O hydrogen bonds, generating chains along [100].

## Related literature

For synthesis of *N*-dimethyl­carbamoyl-*N′*,*N′*,*N′′*,*N′′*-tetra­methyl­guanidine, see: Möllers *et al.* (2003 ▶). For the crystal structures of 2- and 5-azido-*N*-(diphenyl­carbamo­yl) proline methyl ester, see: Lynch *et al.* (1995 ▶).
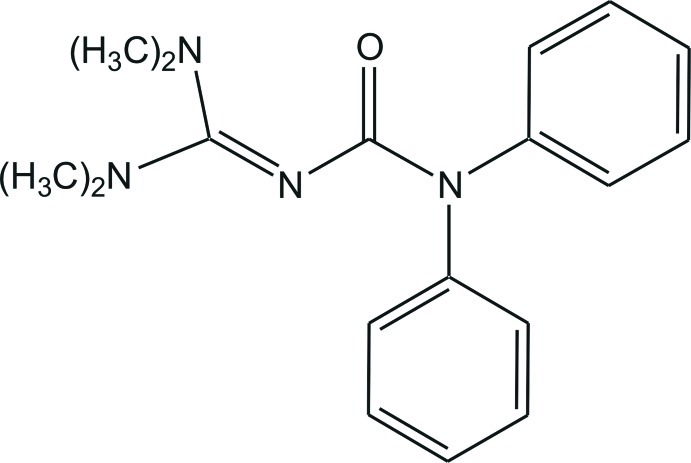


## Experimental

### 

#### Crystal data

C_18_H_22_N_4_O
*M* *_r_* = 310.40
Monoclinic, 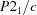

*a* = 7.9321 (3) Å
*b* = 17.0477 (9) Å
*c* = 12.2151 (6) Å
β = 98.583 (2)°
*V* = 1633.28 (13) Å^3^
*Z* = 4
Mo *K*α radiation
μ = 0.08 mm^−1^
*T* = 100 K
0.25 × 0.20 × 0.15 mm

#### Data collection

Bruker Kappa APEXII Duo diffractometer
51490 measured reflections
4990 independent reflections
4200 reflections with *I* > 2σ(*I*)
*R*_int_ = 0.029

#### Refinement

*R*[*F* ^2^ > 2σ(*F* ^2^)] = 0.039
*wR*(*F* ^2^) = 0.105
*S* = 1.04
4990 reflections
212 parameters
H-atom parameters constrained
Δρ_max_ = 0.37 e Å^−3^
Δρ_min_ = −0.23 e Å^−3^

### 

Data collection: *APEX2* (Bruker, 2008 ▶); cell refinement: *SAINT* (Bruker, 2008 ▶); data reduction: *SAINT*; program(s) used to solve structure: *SHELXS97* (Sheldrick, 2008 ▶); program(s) used to refine structure: *SHELXL97* (Sheldrick, 2008 ▶); molecular graphics: *DIAMOND* (Brandenburg & Putz, 2005 ▶); software used to prepare material for publication: *SHELXL97*.

## Supplementary Material

Crystal structure: contains datablock(s) I, global. DOI: 10.1107/S160053681204127X/im2403sup1.cif

Structure factors: contains datablock(s) I. DOI: 10.1107/S160053681204127X/im2403Isup2.hkl

Supplementary material file. DOI: 10.1107/S160053681204127X/im2403Isup3.cml

Additional supplementary materials:  crystallographic information; 3D view; checkCIF report

## Figures and Tables

**Table 1:** Hydrogen-bond geometry (Å, °)

*D*—H⋯*A*	*D*—H	H⋯*A*	*D*⋯*A*	*D*—H⋯*A*
C11—H11*A*⋯O1^i^	0.95	2.59	3.3893 (12)	141

Symmetry code: (i) 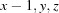.

**Fig. 1.**
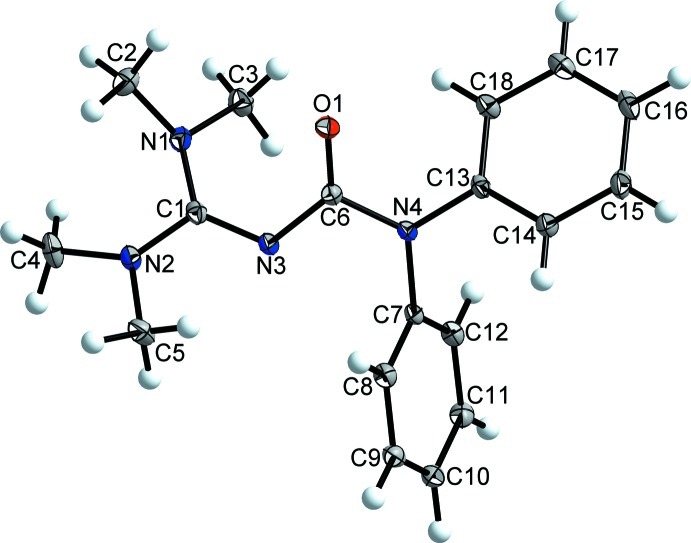
Molecular structure of the title compound with displacement ellipsoids at the 50% probability level.

**Fig. 2.**
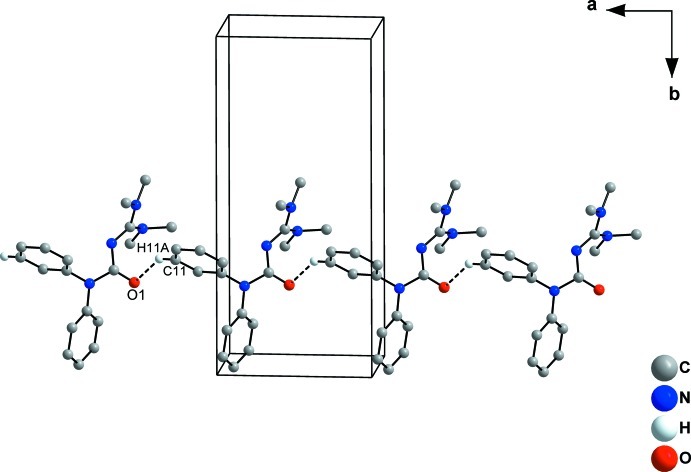
C–H···O hydrogen bonds between the molecules, ab-view. Hydrogen bonds are indicated by dashed lines.

## supplementary crystallographic information

### Comment

3-[bis(dimethylamino)methylene]-1,1-diphenylurea - also known as *N*-diphenylcarbamoyl-*N'*,*N'*,*N''*,*N''*- tetramethylguanidine - is a guanidine derivative bearing an additional urea moiety. Similar to 3-[bis(dimethylamino)methylene]-1,1-dimethylurea (*N*-dimethylcarbamoyl- *N'*,*N'*,*N''*,*N''*-tetramethylguanidine; Möllers *et al.*, 2003), it can be used as a ligand in coordination chemistry to coordinate transition metals through one imino nitrogen and one carbonyl oxygen atom. Therefore, it proved to be important to determine the hitherto unknown crystal structure of the free ligand, to enable comparative investigations. According to the structure analysis, the C1–N3 bond in the title compound is 1.3179 (11) Å, indicating double bond character. The bond lengths C1–N2 = 1.3551 (11) Å and C1–N1 = 1.3737 (11) Å are elongated and characteristic for C_imine_–N_amine_ single bonds. The N–C1–N angles are: 115.91 (8)° (N1–C1–N2), 118.20 (8)° (N2–C1–N3) and 125.69 (8)° (N1–C1–N3), showing a deviation of the CN_3_ plane from an ideal trigonal-planar geometry. Bonds between N atoms and terminal *C*-methyl groups all have values close to typical single bonds (1.4529 (12)–1.4624 (12) Å). The C–O bond length in the diphenylcarbamoyl group is C6–O1 = 1.2305 (11) Å, and shows the expected double-bond character. The N–C bond lengths in the carbamoyl moiety are: N3–C6 = 1.3722 (11) Å, N4–C6 = 1.4028 (11) Å, N4–C13 = 1.4266 (11) Å and N4–C7 = 1.4367 (11) Å. They agree very well with X-ray structural data of the compounds 2- and 5-azido-*N*- (diphenylcarbamoyl)proline methyl ester (Lynch *et al.*, 1995). The dihedral angle C1–N3–C6–N4 is -161.69 (8)° and the angle between the planes N1/C1/N2 and O1/C6/N4 is 51.68 (8)°, which shows a significant twisting of the diphenylcarbamoyl group relative to the CN_3_ plane (Fig. 1). Weak C–H···O hydrogen bonds between aromatic hydrogen atoms and carbonyl oxygen atoms of neighboring molecules have been determined [*d*(H···O) = 2.59 Å] (Tab. 1), generating a chain along the *ab*-plane (Fig. 2). On the other hand, intermolecular C–H···N hydrogen bonds play no prominent role in the stabilization of the crystal structure.

### Experimental

The title compound was obtained by heating two equivalents (60.4 mmol) of *N'*,*N'*,*N''*,*N''*-tetramethylguanidine with one equivalent (30.2 mmol) *N*,*N*-diphenylcarbamoyl chloride in acetonitrile for three hours under reflux. After cooling to room temperature the precipitated *N'*,*N'*,*N''*,*N''*-tetramethylguanidinium chloride was filtered off and the solvent was removed. The residue was redissolved in diethylether and the insoluble part was filtered off. After evaporation of the solvent a colorless solid was been obtained. The title compound crystallized from a saturated acetonitrile solution after several days at 273 K, forming colorless single crystals. Yield: 7.6 g (81%)

### Refinement

The hydrogen atoms of the methyl groups were allowed to rotate with a fixed angle around the C–N bond to best fit the experimental electron density, with *U*_iso_(H) set to 1.5 *U*_eq_(C) and d(C—H) = 0.98 Å. H atoms of the aromatic rings were placed in calculated positions with (C—H) = 0.95 Å. They were included in the refinement using the riding model approximation, with *U*_iso_(H) set to 1.2 *U*_eq_(C).

### Crystal data

**Table:**

C_18_H_22_N_4_O	*F*(000) = 664
*M**_r_* = 310.40	*D*_x_ = 1.262 Mg m^−^^3^
Monoclinic, *P*2_1_/*c*	Melting point: 427 K
Hall symbol: -P 2ybc	Mo *K*α radiation, λ = 0.71073 Å
*a* = 7.9321 (3) Å	Cell parameters from 4990 reflections
*b* = 17.0477 (9) Å	θ = 2.1–30.5°
*c* = 12.2151 (6) Å	µ = 0.08 mm^−^^1^
β = 98.583 (2)°	*T* = 100 K
*V* = 1633.28 (13) Å^3^	Block, colorless
*Z* = 4	0.25 × 0.20 × 0.15 mm

### Data collection

**Table:**

Bruker Kappa APEXII Duo diffractometer	4200 reflections with *I* > 2σ(*I*)
Radiation source: sealed tube	*R*_int_ = 0.029
Graphite monochromator	θ_max_ = 30.5°, θ_min_ = 2.1°
φ scans, and ω scans	*h* = −11→11
51490 measured reflections	*k* = −24→24
4990 independent reflections	*l* = −17→16

### Refinement

**Table:**

Refinement on *F*^2^	Primary atom site location: structure-invariant direct methods
Least-squares matrix: full	Secondary atom site location: difference Fourier map
*R*[*F*^2^ > 2σ(*F*^2^)] = 0.039	Hydrogen site location: difference Fourier map
*wR*(*F*^2^) = 0.105	H-atom parameters constrained
*S* = 1.04	*w* = 1/[σ^2^(*F*_o_^2^) + (0.0541*P*)^2^ + 0.4544*P*] where *P* = (*F*_o_^2^ + 2*F*_c_^2^)/3
4990 reflections	(Δ/σ)_max_ < 0.001
212 parameters	Δρ_max_ = 0.37 e Å^−^^3^
0 restraints	Δρ_min_ = −0.23 e Å^−^^3^

### Special details

**Table:**

Geometry. All e.s.d.'s (except the e.s.d. in the dihedral angle between two l.s. planes) are estimated using the full covariance matrix. The cell e.s.d.'s are taken into account individually in the estimation of e.s.d.'s in distances, angles and torsion angles; correlations between e.s.d.'s in cell parameters are only used when they are defined by crystal symmetry. An approximate (isotropic) treatment of cell e.s.d.'s is used for estimating e.s.d.'s involving l.s. planes.
Refinement. Refinement of *F*^2^ against ALL reflections. The weighted *R*-factor *wR* and goodness of fit *S* are based on *F*^2^, conventional *R*-factors *R* are based on *F*, with *F* set to zero for negative *F*^2^. The threshold expression of *F*^2^ > σ(*F*^2^) is used only for calculating *R*-factors(gt) *etc*. and is not relevant to the choice of reflections for refinement. *R*-factors based on *F*^2^ are statistically about twice as large as those based on *F*, and *R*- factors based on ALL data will be even larger.

### Fractional atomic coordinates and isotropic or equivalent isotropic displacement parameters (Å^2^)

**Table:**

	*x*	*y*	*z*	*U*_iso_*/*U*_eq_	
C1	0.37589 (11)	0.11725 (5)	0.29192 (7)	0.01420 (17)	
N1	0.44747 (10)	0.12889 (5)	0.40021 (7)	0.01682 (16)	
C2	0.63269 (12)	0.12487 (6)	0.43072 (8)	0.0215 (2)	
H2A	0.6820	0.0965	0.3733	0.032*	
H2B	0.6606	0.0973	0.5015	0.032*	
H2C	0.6796	0.1781	0.4379	0.032*	
C3	0.36063 (13)	0.17835 (6)	0.47145 (8)	0.02078 (19)	
H3A	0.3971	0.2329	0.4654	0.031*	
H3B	0.3893	0.1608	0.5484	0.031*	
H3C	0.2371	0.1746	0.4485	0.031*	
N2	0.41847 (10)	0.04945 (4)	0.24502 (7)	0.01680 (16)	
C4	0.47540 (14)	−0.02051 (6)	0.30890 (9)	0.0239 (2)	
H4A	0.4702	−0.0110	0.3874	0.036*	
H4B	0.5930	−0.0327	0.2992	0.036*	
H4C	0.4013	−0.0648	0.2829	0.036*	
C5	0.36459 (14)	0.03644 (6)	0.12745 (8)	0.0218 (2)	
H5A	0.2532	0.0104	0.1162	0.033*	
H5B	0.4483	0.0032	0.0982	0.033*	
H5C	0.3560	0.0869	0.0886	0.033*	
N3	0.26184 (10)	0.16304 (4)	0.23400 (7)	0.01607 (16)	
C6	0.27494 (11)	0.24288 (5)	0.24670 (7)	0.01419 (16)	
O1	0.40355 (8)	0.28089 (4)	0.28186 (6)	0.01803 (14)	
N4	0.12115 (10)	0.28111 (4)	0.20749 (7)	0.01481 (15)	
C7	−0.02172 (11)	0.23987 (5)	0.14776 (7)	0.01345 (16)	
C8	−0.00729 (12)	0.20271 (5)	0.04806 (7)	0.01524 (17)	
H8A	0.0983	0.2033	0.0202	0.018*	
C9	−0.14748 (12)	0.16466 (5)	−0.01074 (8)	0.01697 (18)	
H9A	−0.1369	0.1387	−0.0782	0.020*	
C10	−0.30287 (12)	0.16456 (5)	0.02879 (8)	0.01796 (18)	
H10A	−0.3989	0.1392	−0.0120	0.022*	
C11	−0.31730 (12)	0.20166 (5)	0.12827 (8)	0.01758 (18)	
H11A	−0.4232	0.2014	0.1557	0.021*	
C12	−0.17678 (12)	0.23931 (5)	0.18795 (8)	0.01584 (17)	
H12A	−0.1869	0.2645	0.2560	0.019*	
C13	0.10260 (11)	0.36346 (5)	0.22264 (7)	0.01373 (16)	
C14	0.01332 (12)	0.40811 (5)	0.13736 (8)	0.01772 (18)	
H14A	−0.0281	0.3842	0.0683	0.021*	
C15	−0.01532 (13)	0.48785 (5)	0.15331 (9)	0.0216 (2)	
H15A	−0.0774	0.5178	0.0952	0.026*	
C16	0.04600 (13)	0.52374 (5)	0.25311 (9)	0.0221 (2)	
H16A	0.0259	0.5779	0.2638	0.026*	
C17	0.13735 (13)	0.47939 (5)	0.33744 (8)	0.02066 (19)	
H17A	0.1811	0.5038	0.4057	0.025*	
C18	0.16545 (12)	0.39969 (5)	0.32295 (8)	0.01654 (18)	
H18	0.2274	0.3700	0.3813	0.020*	

### Atomic displacement parameters (Å^2^)

**Table:**

	*U*^11^	*U*^22^	*U*^33^	*U*^12^	*U*^13^	*U*^23^
C1	0.0131 (4)	0.0118 (4)	0.0181 (4)	−0.0006 (3)	0.0039 (3)	0.0001 (3)
N1	0.0154 (4)	0.0174 (3)	0.0175 (4)	0.0034 (3)	0.0019 (3)	−0.0007 (3)
C2	0.0164 (4)	0.0251 (5)	0.0222 (5)	0.0006 (4)	−0.0001 (3)	0.0032 (4)
C3	0.0236 (5)	0.0201 (4)	0.0195 (4)	0.0025 (4)	0.0061 (4)	−0.0027 (3)
N2	0.0200 (4)	0.0120 (3)	0.0186 (4)	0.0032 (3)	0.0038 (3)	0.0001 (3)
C4	0.0307 (5)	0.0137 (4)	0.0288 (5)	0.0067 (4)	0.0097 (4)	0.0038 (4)
C5	0.0283 (5)	0.0174 (4)	0.0201 (4)	−0.0012 (4)	0.0047 (4)	−0.0049 (3)
N3	0.0155 (4)	0.0105 (3)	0.0212 (4)	0.0007 (3)	−0.0005 (3)	−0.0004 (3)
C6	0.0143 (4)	0.0125 (4)	0.0158 (4)	0.0007 (3)	0.0021 (3)	−0.0006 (3)
O1	0.0147 (3)	0.0144 (3)	0.0244 (3)	−0.0018 (2)	0.0008 (3)	−0.0017 (2)
N4	0.0140 (3)	0.0094 (3)	0.0201 (4)	0.0000 (3)	−0.0005 (3)	−0.0019 (3)
C7	0.0143 (4)	0.0092 (3)	0.0163 (4)	−0.0005 (3)	0.0004 (3)	0.0005 (3)
C8	0.0158 (4)	0.0138 (4)	0.0166 (4)	0.0002 (3)	0.0041 (3)	0.0000 (3)
C9	0.0213 (4)	0.0140 (4)	0.0151 (4)	−0.0003 (3)	0.0011 (3)	−0.0010 (3)
C10	0.0174 (4)	0.0132 (4)	0.0222 (4)	−0.0021 (3)	−0.0008 (3)	0.0004 (3)
C11	0.0146 (4)	0.0157 (4)	0.0229 (4)	−0.0009 (3)	0.0044 (3)	0.0018 (3)
C12	0.0176 (4)	0.0133 (4)	0.0170 (4)	0.0005 (3)	0.0040 (3)	−0.0005 (3)
C13	0.0132 (4)	0.0100 (4)	0.0187 (4)	−0.0005 (3)	0.0045 (3)	−0.0005 (3)
C14	0.0174 (4)	0.0137 (4)	0.0213 (4)	0.0006 (3)	0.0002 (3)	0.0002 (3)
C15	0.0199 (4)	0.0131 (4)	0.0309 (5)	0.0027 (3)	0.0011 (4)	0.0035 (4)
C16	0.0214 (5)	0.0116 (4)	0.0344 (5)	0.0012 (3)	0.0080 (4)	−0.0028 (4)
C17	0.0242 (5)	0.0155 (4)	0.0233 (5)	−0.0017 (3)	0.0070 (4)	−0.0055 (3)
C18	0.0196 (4)	0.0138 (4)	0.0169 (4)	−0.0008 (3)	0.0050 (3)	−0.0004 (3)

### Geometric parameters (Å, º)

**Table:**

C1—N3	1.3179 (11)	N4—C7	1.4367 (11)
C1—N2	1.3551 (11)	C7—C12	1.3907 (13)
C1—N1	1.3737 (11)	C7—C8	1.3925 (12)
N1—C3	1.4566 (12)	C8—C9	1.3909 (12)
N1—C2	1.4624 (12)	C8—H8A	0.9500
C2—H2A	0.9800	C9—C10	1.3893 (14)
C2—H2B	0.9800	C9—H9A	0.9500
C2—H2C	0.9800	C10—C11	1.3897 (14)
C3—H3A	0.9800	C10—H10A	0.9500
C3—H3B	0.9800	C11—C12	1.3940 (13)
C3—H3C	0.9800	C11—H11A	0.9500
N2—C5	1.4529 (12)	C12—H12A	0.9500
N2—C4	1.4594 (12)	C13—C14	1.3949 (12)
C4—H4A	0.9800	C13—C18	1.3961 (12)
C4—H4B	0.9800	C14—C15	1.3967 (13)
C4—H4C	0.9800	C14—H14A	0.9500
C5—H5A	0.9800	C15—C16	1.3857 (15)
C5—H5B	0.9800	C15—H15A	0.9500
C5—H5C	0.9800	C16—C17	1.3910 (14)
N3—C6	1.3722 (11)	C16—H16A	0.9500
C6—O1	1.2305 (11)	C17—C18	1.3923 (12)
C6—N4	1.4028 (11)	C17—H17A	0.9500
N4—C13	1.4266 (11)	C18—H18	0.9500
			
N3—C1—N2	118.20 (8)	C6—N4—C7	121.73 (7)
N3—C1—N1	125.69 (8)	C13—N4—C7	117.30 (7)
N2—C1—N1	115.91 (8)	C12—C7—C8	119.87 (8)
C1—N1—C3	119.63 (8)	C12—C7—N4	119.75 (8)
C1—N1—C2	119.59 (8)	C8—C7—N4	120.34 (8)
C3—N1—C2	114.93 (8)	C9—C8—C7	120.00 (9)
N1—C2—H2A	109.5	C9—C8—H8A	120.0
N1—C2—H2B	109.5	C7—C8—H8A	120.0
H2A—C2—H2B	109.5	C10—C9—C8	120.24 (8)
N1—C2—H2C	109.5	C10—C9—H9A	119.9
H2A—C2—H2C	109.5	C8—C9—H9A	119.9
H2B—C2—H2C	109.5	C9—C10—C11	119.76 (8)
N1—C3—H3A	109.5	C9—C10—H10A	120.1
N1—C3—H3B	109.5	C11—C10—H10A	120.1
H3A—C3—H3B	109.5	C10—C11—C12	120.20 (9)
N1—C3—H3C	109.5	C10—C11—H11A	119.9
H3A—C3—H3C	109.5	C12—C11—H11A	119.9
H3B—C3—H3C	109.5	C7—C12—C11	119.92 (8)
C1—N2—C5	119.54 (8)	C7—C12—H12A	120.0
C1—N2—C4	123.21 (8)	C11—C12—H12A	120.0
C5—N2—C4	115.19 (8)	C14—C13—C18	119.28 (8)
N2—C4—H4A	109.5	C14—C13—N4	119.39 (8)
N2—C4—H4B	109.5	C18—C13—N4	121.27 (8)
H4A—C4—H4B	109.5	C13—C14—C15	120.13 (9)
N2—C4—H4C	109.5	C13—C14—H14A	119.9
H4A—C4—H4C	109.5	C15—C14—H14A	119.9
H4B—C4—H4C	109.5	C16—C15—C14	120.62 (9)
N2—C5—H5A	109.5	C16—C15—H15A	119.7
N2—C5—H5B	109.5	C14—C15—H15A	119.7
H5A—C5—H5B	109.5	C15—C16—C17	119.15 (9)
N2—C5—H5C	109.5	C15—C16—H16A	120.4
H5A—C5—H5C	109.5	C17—C16—H16A	120.4
H5B—C5—H5C	109.5	C16—C17—C18	120.79 (9)
C1—N3—C6	119.50 (8)	C16—C17—H17A	119.6
O1—C6—N3	127.35 (8)	C18—C17—H17A	119.6
O1—C6—N4	120.53 (8)	C17—C18—C13	120.02 (9)
N3—C6—N4	112.02 (7)	C17—C18—H18	120.0
C6—N4—C13	120.94 (7)	C13—C18—H18	120.0
			
N3—C1—N1—C3	21.15 (14)	C12—C7—C8—C9	−0.43 (13)
N2—C1—N1—C3	−153.60 (9)	N4—C7—C8—C9	−178.31 (8)
N3—C1—N1—C2	−130.58 (10)	C7—C8—C9—C10	0.95 (13)
N2—C1—N1—C2	54.67 (12)	C8—C9—C10—C11	−0.94 (13)
N3—C1—N2—C5	11.89 (13)	C9—C10—C11—C12	0.41 (14)
N1—C1—N2—C5	−172.95 (8)	C8—C7—C12—C11	−0.09 (13)
N3—C1—N2—C4	−151.00 (9)	N4—C7—C12—C11	177.79 (8)
N1—C1—N2—C4	24.16 (13)	C10—C11—C12—C7	0.11 (13)
N2—C1—N3—C6	−146.79 (9)	C6—N4—C13—C14	140.27 (9)
N1—C1—N3—C6	38.57 (14)	C7—N4—C13—C14	−37.84 (12)
C1—N3—C6—O1	22.08 (15)	C6—N4—C13—C18	−42.63 (13)
C1—N3—C6—N4	−161.69 (8)	C7—N4—C13—C18	139.26 (9)
O1—C6—N4—C13	−9.58 (13)	C18—C13—C14—C15	−1.21 (14)
N3—C6—N4—C13	173.89 (8)	N4—C13—C14—C15	175.95 (9)
O1—C6—N4—C7	168.45 (8)	C13—C14—C15—C16	0.78 (15)
N3—C6—N4—C7	−8.08 (12)	C14—C15—C16—C17	0.25 (15)
C6—N4—C7—C12	121.44 (9)	C15—C16—C17—C18	−0.84 (15)
C13—N4—C7—C12	−60.46 (11)	C16—C17—C18—C13	0.40 (15)
C6—N4—C7—C8	−60.69 (12)	C14—C13—C18—C17	0.63 (14)
C13—N4—C7—C8	117.41 (9)	N4—C13—C18—C17	−176.47 (9)

### Hydrogen-bond geometry (Å, º)

**Table:**

*D*—H···*A*	*D*—H	H···*A*	*D*···*A*	*D*—H···*A*
C11—H11*A*···O1^i^	0.95	2.59	3.3893 (12)	141

Symmetry code: (i) *x*−1, *y*, *z*.
